# Protein-Based Systems for Translational Regulation of Synthetic mRNAs in Mammalian Cells

**DOI:** 10.3390/life11111192

**Published:** 2021-11-05

**Authors:** Hideyuki Nakanishi

**Affiliations:** Department of Biofunction Research, Institute of Biomaterials and Bioengineering, Tokyo Medical and Dental University (TMDU), 2-3-10 Kanda-Surugadai, Chiyoda-ku, Tokyo 101-0062, Japan; nakanishi.hideyuki.3m@kyoto-u.jp

**Keywords:** synthetic biology, messenger RNA, RNA binding protein, translation, modified nucleoside

## Abstract

Synthetic mRNAs, which are produced by in vitro transcription, have been recently attracting attention because they can express any transgenes without the risk of insertional mutagenesis. Although current synthetic mRNA medicine is not designed for spatiotemporal or cell-selective regulation, many preclinical studies have developed the systems for the translational regulation of synthetic mRNAs. Such translational regulation systems will cope with high efficacy and low adverse effects by producing the appropriate amount of therapeutic proteins, depending on the context. Protein-based regulation is one of the most promising approaches for the translational regulation of synthetic mRNAs. As synthetic mRNAs can encode not only output proteins but also regulator proteins, all components of protein-based regulation systems can be delivered as synthetic mRNAs. In addition, in the protein-based regulation systems, the output protein can be utilized as the input for the subsequent regulation to construct multi-layered gene circuits, which enable complex and sophisticated regulation. In this review, I introduce what types of proteins have been used for translational regulation, how to combine them, and how to design effective gene circuits.

## 1. Introduction

During gene expression, genes are first transcribed from DNA to messenger RNA (mRNA) and then translated from mRNA to protein. Thus, we can make cells express exogenous genes by transfecting either DNA or mRNA. Although DNA transfection is the standard method for transgene expression, it can cause insertional mutagenesis of endogenous genes, which is a major drawback in medical applications. In contrast, synthetic mRNAs, which are produced by in vitro transcription, can be used for transgene expression without the risk of insertional mutagenesis. Thus, synthetic mRNAs have recently been attracting attention as tools for gene therapy, cellular reprogramming, and vaccine development [1]. 

Nevertheless, context-dependent regulation of transgene expression is more difficult in synthetic mRNA transfection than in DNA transfection. Upon DNA transfection, transgene expression can be tuned using transcriptional regulatory sequences, such as drug-inducible or tissue-specific promoters. On the other hand, such transcriptional regulation cannot apply to synthetic mRNA transfection as they must be transcribed in vitro prior to transfection. As some therapeutic genes may cause adverse effects when they are expressed in inappropriate contexts, context-dependent transgene regulation is helpful to cope with low adverse effects and high therapeutic efficacy. For example, cell-selective expression of pro-apoptotic genes [2,3,4,5,6] may enable cancer cell elimination without damaging healthy cells. Similarly, cardiomyocyte-selective expression of proliferation-promoting genes can be helpful to cope with myocardial regeneration without elevating fibrosis and immune response [7]. Thus, the development of translational regulation systems will make synthetic mRNAs more valuable.

**Figure 1:**
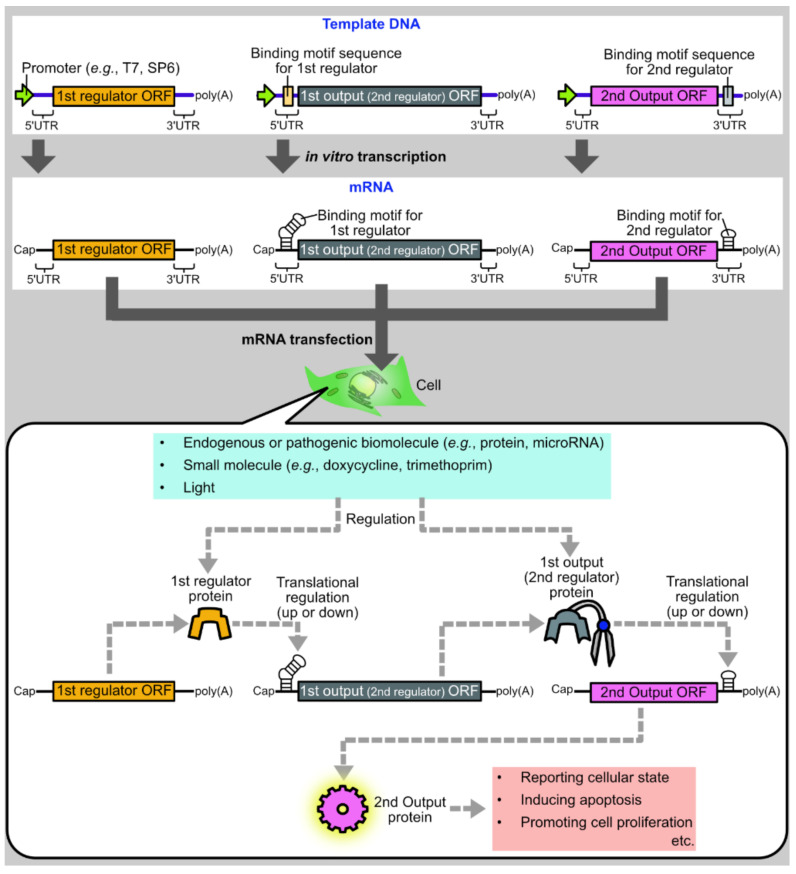
Schematic diagram of a protein-based translational regulation system for synthetic mRNAs. Synthetic mRNAs encoding regulator or output proteins are synthesized by in vitro transcription and transfected into cells. After transfection, a regulator protein is translated from an mRNA and regulates the translation of another mRNA encoding an output protein. Then, the output protein induces various biological phenomena such as apoptosis or cell proliferation. In multi-layered gene circuits, the 1st output protein acts as the 2nd regulator to regulate the translation of the 2nd output ORF. For cell state-responsive autonomous regulation, regulator proteins or mRNAs are designed to respond to endogenous or pathogenic biomolecules such as proteins or microRNAs. Regulator proteins can also be designed to respond to external cues such as small molecules or light for deliberate control of translation.

One promising approach is to develop protein-based systems for translational regulation of synthetic mRNAs. There are multiple advantages to protein-based translational regulation systems. First, when proteins are used as translational regulators, the regulators themselves can be translated from synthetic mRNAs, such that all components necessary for regulation can be delivered as synthetic mRNAs. Second, compared to ribozyme-embedded mRNAs [8] or caged mRNAs [9], mRNAs composing protein-based regulation systems have less concern regarding the alteration of mRNA properties during synthesis or storage, such as self-cleavage or decaging. Finally, in protein-based translational regulation systems, the first output protein can be used as the input for the second regulation, allowing layered gene circuits for sophisticated regulation (Figure 1). 

In this review, I introduce protein-based systems for the translational regulation of synthetic mRNAs in mammalian cells.

## 2. Basic Protein Modules for Protein-Based Translational Regulation Systems

### 2.1. Binding to Target mRNAs

**Figure 2:**
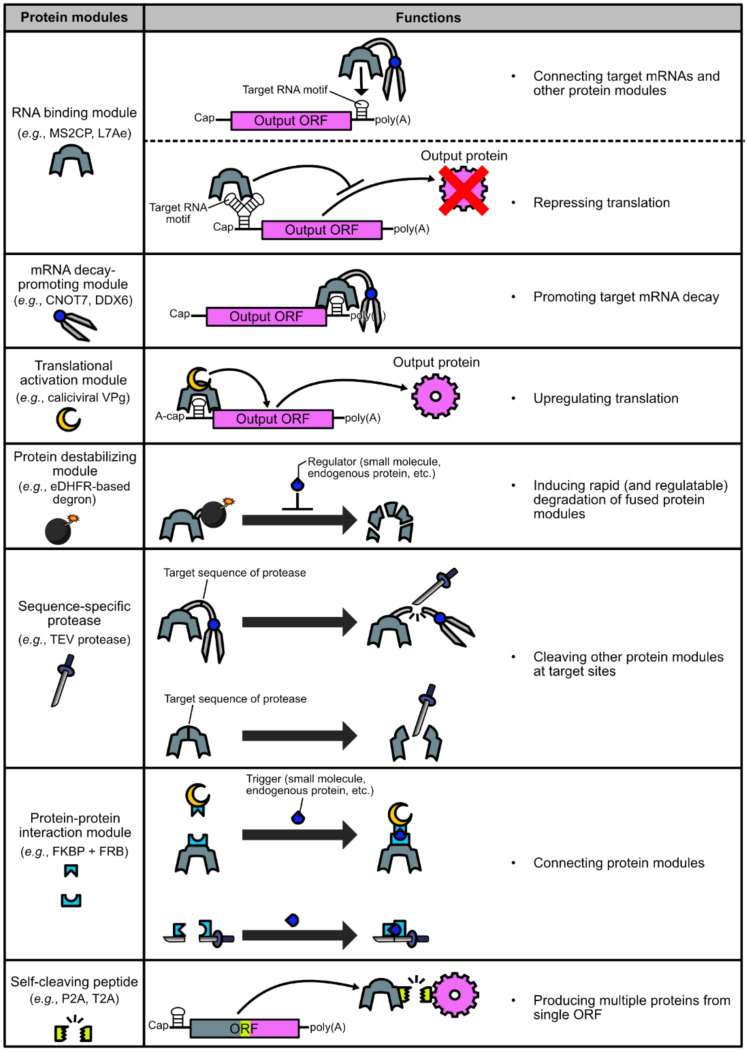
List of protein modules used in translational regulation of synthetic mRNAs.

The most fundamental modules of protein-based translational regulation systems are proteins that bind specifically to RNA motifs on synthetic mRNAs that need to be regulated. Motif-specific RNA binding proteins (RBPs) are mainly used for two purposes. One is to fuse RBPs with other protein modules, so that other modules can act on synthetic mRNAs. The other is to repress translation only by the binding of RBPs to the 5' untranslated regions (UTRs) of synthetic mRNAs, without being combined with other protein modules (Figure 2, the 1st row). 

**Figure 3:**
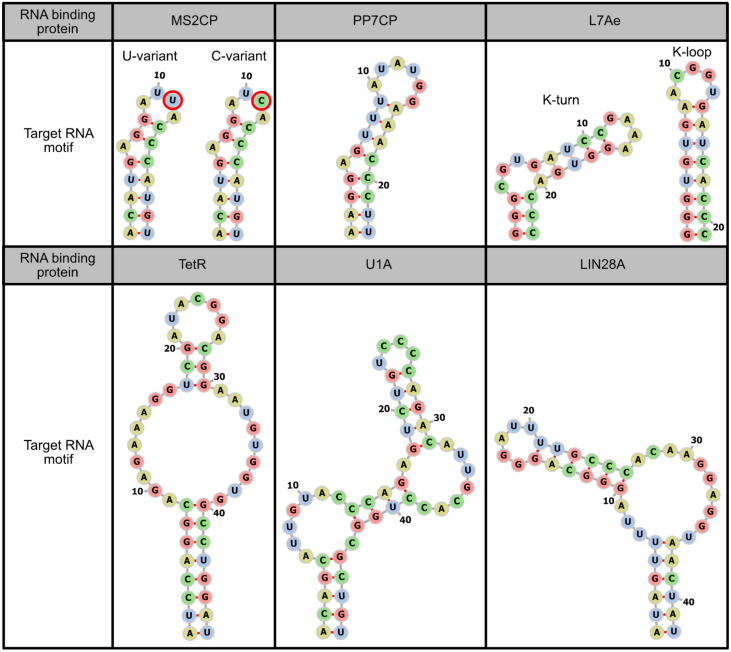
Target RNA motifs of representative RNA binding proteins. Sequences and secondary structures of target RNA motifs of MS2CP [28], PP7CP [21], L7Ae [11], TetR [26], U1A [29], and LIN28A [27] are shown. The secondary structures are visualized by forna [30].

In order to regulate the translation of only synthetic mRNAs while avoiding the effect on endogenous mRNAs, it is desirable to use RBPs that have high specificity for target RNA motifs, and these motifs are not present in endogenous mRNAs. Thus, microbial RBPs are primarily used in mRNA-based mammalian synthetic biology. The representative microbial RBPs used to regulate synthetic mRNAs are coat proteins derived from the bacteriophages MS2 (MS2CP) [2,3,4,5,6,10,11,12,13,14,15,16,17,18,19,20,21] and PP7 (PP7CP) [6,14,21], the archaeal ribosomal protein L7Ae [2,3,4,6,7,11,12,21,22,23,24,25], and the tetracycline-responsive repressor protein (TetR) from *Escherichia coli* [23,26]. Among these, TetR is unique in its ability to conditionally dissociate from the target RNA motif by doxycycline addition. In addition to these microbial proteins, mammalian RBPs can also be used, such as U1A, a spliceosomal protein [10,13,21,27], and LIN28A, a pre-microRNA binding protein [21,27] (Figure 3). However, when these mammalian proteins and their target RNA motifs are used in mammalian cells, it should be noted that they may interact with endogenous RNAs or proteins. Such interactions can induce unintended effects on both the regulation of synthetic mRNAs and endogenous pathways.

### 2.2. Promoting Target mRNA Decay

Fusion of motif-specific RBPs and mRNA decay-promoting proteins, such as dead box helicase 6 (DDX6) [23] and CCR4-NOT transcription complex subunit 7 (CNOT7) [2,12,14], are used to induce targeted mRNA decay and eventual translational shut-off. DDX6 is a protein that interacts with decapping coactivators and the CCR4-NOT complex, which has a role in mRNA deadenylation and translational repression [31], whereas CNOT7 is a deadenylase module of the CCR4-NOT complex [32]. Since 5’ cap structures and poly(A) tails have a crucial role in both mRNA stability and translation, targeted binding of these proteins to synthetic mRNAs can promote their decay and translational shut-off (Figure 2, the 2nd row). In addition, studies using pDNA transfection reported that mRNA decay can also be promoted by targeted binding of nonsense-mediated mRNA decay (NMD)-related proteins (e.g., Y14, RNPS1, and Upf1, 2, 3a, and 3b) [16,17,18,19], Staufen1, which can induce Staufen-mediated mRNA decay (SMD) [16,17], and the RNAi-related protein Ago2 [33]. 

Although translational repression can be achieved by only RBPs, combining mRNA decay-promoting proteins has two advantages. First, in the case that an RBP itself cannot sufficiently repress translation, the fusion of an mRNA decay-promoting protein may be helpful to improve the translational repression efficiency. Wagner et al. reported that the TetR-DDX6 fusion protein can be used for doxycycline-controllable translational repression of an N1-methyl-pseudouridine-containing mRNA, whereas TetR only cannot [23]. The second advantage of combining mRNA decay-promoting proteins is that the insertion sites of target RNA motifs can be placed in 3’ UTRs [2,12,14,16,17,18,19,33]. In the case of translational repression using only RBPs, target motifs need to be inserted in 5' UTRs, but the presence of stable stem structures in 5' UTRs is itself disadvantageous for translation [34]. Therefore, designing mRNAs for high protein production efficiency is relatively easy when target motifs are placed in 3’ UTRs instead of 5’ UTRs.

It is stated that the regulation by CNOT7 is more effective in RNA replicons than in non-replicative mRNAs [6,14]. RNA replicons, which are also called self-amplifying mRNAs, replicate themselves in the cytoplasm through the activity of RNA-dependent RNA polymerase (also called RNA replicase) that is expressed from RNA replicons themselves. Thus, while the amount of protein produced by conventional synthetic mRNAs decreases over time due to mRNA decay by the endogenous machinery, RNA replicons replenish themselves and enable continuous protein production by single transfection [35,36]. Nevertheless, caution is required when mRNA decay-promoting proteins are combined with RNA replicons. If RNA replicons are completely removed by mRNA decay-promoting proteins, the replenishment of RNA replicons cannot occur. Although there is a report that RNA replicons can partially be restored after the termination of promoted decay [14], designing RNA replicon-based gene circuits containing mRNA decay-promoting proteins should be done with caution, especially when there is a long duration of promoted mRNA decay.

### 2.3. Activating Target mRNA Translation

Translation of a eukaryotic mRNA is typically initiated by the binding of the eukaryotic initiation factor (eIF) 4F complex to the cap structure at the 5' end of the mRNA, which in turn recruits the 40S subunit of the ribosome via the eIF4F-eIF3-40S subunit interaction [37]. Therefore, two components are needed to achieve conditional activation of mRNA translation by protein-based systems. One is an mRNA lacking the canonical 5' cap (typically, a translationally inactive cap analog, termed "A-Cap", is used instead of the canonical cap), and the other is a translational activator protein that can directly or indirectly recruit ribosomes to mRNAs even in the absence of the canonical 5' cap.

One of the proteins that can recruit ribosomes is the viral protein genome-linked (VPg) from calicivirus. Caliciviral VPg is a relatively small (e.g., 111 amino acids in the case of feline calicivirus) protein that is fused to the 5' end of the caliciviral RNA. Since caliciviral RNAs lack the 5' cap, caliciviruses use VPg to recruit ribosomes to their RNAs via a VPg-eIF4F interaction [38,39,40]. We showed that the fusion protein of MS2CP and the feline caliciviral VPg, named caliciviral VPg-based translational activator (CaVT), can activate the translation of A-capped mRNAs containing the MS2CP-target motif in their 5' UTRs [5,20] (Figure 2, the 3rd row).

Another example of a translational activator protein is a fusion protein composed of MS2CP and the C-terminal region (amino acids 623-1600) of eIF4G. eIF4G is a component of the eIF4F complex, and its C-terminal region has a role in the interaction with eIF3 and the 40S subunit of the ribosome. Paek et al. reported that connecting the C-terminal region of eIF4G to the 3’ UTRs of A-capped mRNAs can activate their translation [15]. It should be noted, however, that in the study, the MS2CP-eIF4G fusion protein was expressed not from a synthetic mRNA, but a cytomegalovirus promoter-embedded plasmid DNA (pDNA). Compared to synthetic mRNA, pDNA containing a strong promoter shows a higher maximum level of expression, although the variability in the expression level among cells is also higher [41]. Therefore, future studies are needed to determine whether synthetic mRNAs can produce an adequate amount of MS2CP-eIF4G to activate the translation of A-capped mRNAs. The translational activating function of eIF4E, another component of the eIF4F complex, was also shown in experiments using pDNA transfection [42].

### 2.4. Destabilizing Proteins

Destabilizing domains (also called degrons) that induce rapid degradation of fused proteins are used to regulate the abundance of translational regulator proteins (Figure 2, the 4th row). For example, rapid degradation of a translational repressor protein results in an enhancement of its target mRNA translation [4,6,14,20,23,25]. Conversely, rapid degradation of a translational activator protein diminishes the translation of its target mRNA [20]. In many cases, the purpose is to achieve deliberate or cell state-responsive translational regulation by using degrons whose destabilizing activity can be altered by small molecules or endogenous biomolecules. 

There are several small molecule-responsive degrons, such as the FK506-binding protein (FKBP)-based degron [43] and the auxin-inducible degron [44], and the *Escherichia coli*-derived dihydrofolate reductase (eDHFR)-based degron [45] is one of the most popular. Importantly, the destabilizing activity of the eDHFR-based degron can be inhibited by trimethoprim, an antibiotic that is used clinically. Translational regulator proteins fused with the eDHFR-based degron are rapidly degraded in the absence of trimethoprim, but can be stabilized by the addition of trimethoprim, allowing them to repress or activate the translation of their target mRNAs [4,6,20,23,25]. In addition, we recently showed that the combination of the eDHFR-based degron and photocaged trimethoprim, which can bind the degron only after light irradiation, enables photo-regulatable translation [20]. 

**Figure 4:**
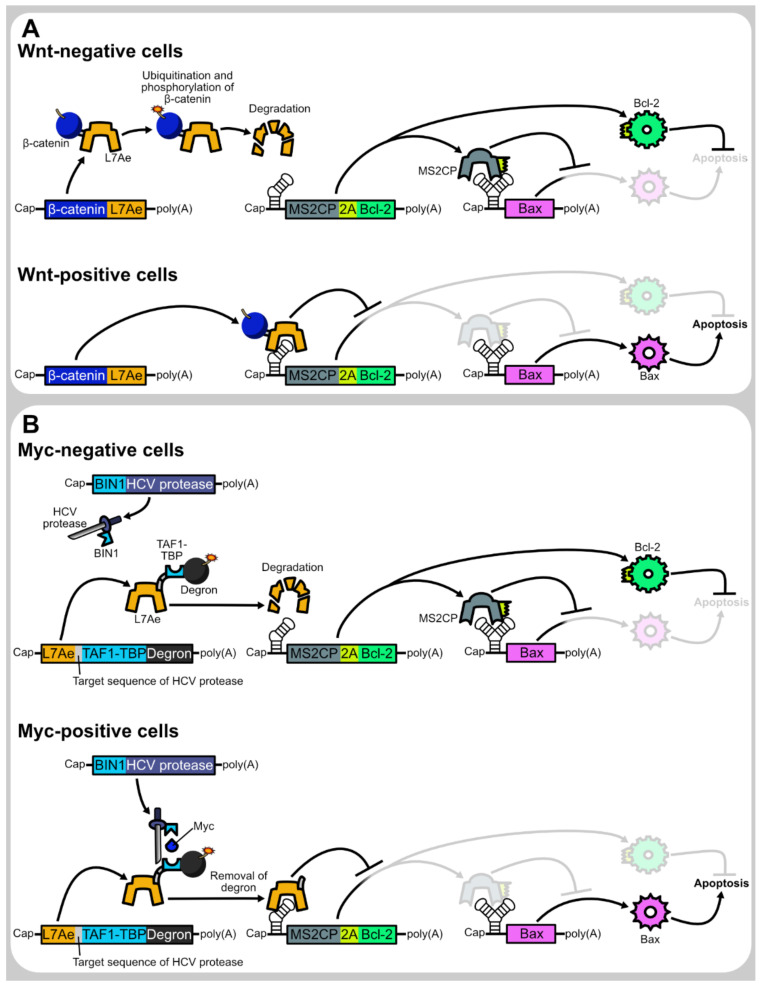
Representatives of endogenous protein-responsive translational regulation systems. (**A**) Wnt-responsive apoptosis induction system. In Wnt-negative cells, β-catenin-fused L7Ae is rapidly degraded due to ubiquitination and phosphorylation of β-catenin. Therefore, the L7Ae target motif (K-turn)-embedded mRNA encoding MS2CP and Bcl-2 is not translationally repressed. The translated MS2CP represses the translation of Bax, whereas Bcl-2 prevents apoptosis caused by leaky expression of Bax (top). In contrast, in Wnt-positive cells, β-catenin-fused L7Ae is not rapidly degraded and represses the translation of MS2CP and Bcl-2. As MS2CP translation is repressed, Bax is not translationally repressed by MS2CP and induces apoptosis (bottom). (**B**) Myc-responsive apoptosis induction system. In Myc-negative cells, L7Ae is rapidly degraded due to the fused degron. Similar to the case of the Wnt-responsive system shown above, the rapid degradation of L7Ae results in the prevention of apoptosis (top). In Myc-positive cells, TAF1-TBP that is fused to L7Ae attracts BIN1-fused HCV protease. Then, HCV protease cleaved its target sequence inserted between L7Ae and TAF1-TBP-degron. Due to the cleavage, the degron is removed from L7Ae, which stabilizes L7Ae. Then, stabilized L7Ae translationally represses MS2CP and Bcl-2, thereby induces Bax-mediated apoptosis (bottom).

In mammalian cells, the degradation of some proteins is known to be promoted in response to cell state, and such proteins can be used as cell state-responsive degrons. One example used in translational regulation is β-catenin, which becomes a target for ubiquitination and phosphorylation to promote degradation in Wnt-negative cells. Utilizing this feature of β-catenin, Yang and Ding achieved Wnt-positive cell-selective translational repression by the β-catenin-fused translational repressor protein [4] (Figure 4A). They also used another type of degron whose destabilizing activity is activated when its N-terminal region is removed by a sequence-specific protease [4,6,46]. Therefore, the protease can relieve mRNAs from translational repression caused by the degron-fused translational repressor protein.

### 2.5. Cleaving Proteins

Some viruses such as tobacco etch virus (TEV), tobacco vein mottling virus (TVMV) [47], and hepatitis C virus (HCV) [48] have sequence-specific proteases that recognize and cleave the target sequences of five to seven amino acid residues. When multiple proteins are connected by a linker containing a target sequence of such protease, the protease can separate these proteins and alter the property of the fusion construct (Figure 2, the 5th row). One example is abolishing promoted mRNA decay by separating the RNA binding module and the mRNA decay-promoting module [12,14]. A fusion protein consisting of an RBP (MS2CP or PP7CP) and CNOT7 can normally promote the decay of mRNAs that have target RNA motifs. However, if the linker connecting these two proteins contains a target sequence of protease, the corresponding protease can separate CNOT7 from the RBP, thereby abolishing CNOT7-mediated mRNA decay. Similarly, when a protease target sequence is inserted between the degron and the translational regulator protein, the corresponding protease can stabilize the translational regulator by removing the degron from it [4,6]. 

If a target sequence can be inserted without impairing the function of the protein, sequence-specific proteases can also be used to cleave the protein that is not constructed by fusing multiple proteins. For example, Cella et al. showed that L7Ae with a TEV protease target sequence can repress translation in the absence of TEV protease, and translational repression can be released by cleavage of L7Ae by TEV protease [12]. 

When multiple proteases are used simultaneously in a single system, the orthogonality of these proteases needs to be checked, as unexpected cleavage by non-corresponding proteases can cause unexpected output. For example, Cella et al. reported that the turnip mosaic virus (TUMV) protease has moderate cleavage activity against the target sequences of TEV, TVMV, and sunflower mosaic virus (SuMMV) [12].

### 2.6. Combining Separate Proteins

Protein–protein interaction modules that respond to specific cues (e.g., small molecules or light) are useful to achieve deliberate or cell state-responsive autonomous translational regulation. Representative protein–protein interaction modules are FKBP-FRB and its variant [5,6,14,49,50], ABI-PLY [4,6,14], and CRY2-CIBN [6,14,51]. By fusing such protein–protein interaction modules to two proteins with different functions, one can control the effect on mRNAs or other protein modules (Figure 2, the 6th row). One example is our small molecule-controllable translational activation system. By fusing small molecule-responsive hetero-dimerization domains to MS2CP and VPg, we have succeeded in inducing translational activation in the small molecule-dependent manner [5,20]. Furthermore, the addition of a photo-cage to such a trigger molecule enabled photo-controllable translational activation [20]. Similarly, intracellular proteins can also be used as triggers to control protein–protein interactions. Yang and Ding reported the system that selectively induces translational repression in Myc-positive cells [4]. This system is composed of two fusion proteins. One is an engineered translational repressor protein composed of L7Ae, an HCV protease target sequence, TAF1-TBP, and a degron. The other is an HCV protease fused with BIN1. As both BIN1 and TAF1-TBP bind Myc, in Myc-positive cells, the BIN1-fused HCV protease is attracted to the engineered translational repressor by the BIN1–Myc–TAF1-TBP interaction. Then, HCV protease removes the degron from the translational repressor to abolish the degron-induced destabilization of L7Ae. Thus, target mRNA translation was repressed in Myc-positive cells (Figure 4B). Another example is the selective translation system for viral protein-expressing cells, using a translational repressor containing a TEV protease target sequence [12]. In that study, Cella et al. designed two fusion proteins. One is an intrabody-fused L7Ae which has a cleavage sequence of TEV protease. The other is an intrabody-fused TEV protease. Both intrabodies bind the viral protein NS3, but their target epitopes are different. Thus, in cells expressing NS3, the TEV protease is attracted to L7Ae via the intrabody–NS3–intrabody interaction. Then, the TEV protease cleaves L7Ae, thereby abolishing L7Ae-mediated translational repression.

Protein–protein interaction modules can also be used to reconstitute a full-length protein from split fragments. Liu et al. developed various split proteases that are fused with small molecule- or light-responsive protein–protein interaction modules. These split protease fragments do not cleave their target sequences until they interact with one another. The corresponding cues (a small molecule or light) induce the interaction of these split fragments to reconstitute the full-length proteases [14]. When using split proteins, the split-site should be carefully selected because, depending on the split-site, fragments may spontaneously assemble independently of the protein–protein interaction modules or may not function even if it binds via the interaction modules. The tool to predict suitable split sites may be helpful to design split proteins [52].

### 2.7. Producing Multiple Proteins from a Single ORF

Self-cleaving peptides such as porcine teschovirus-1-derived 2A (P2A) [53] can be utilized to link the production of multiple proteins (Figure 2, the 7th row). For example, a translational regulator protein and a fluorescent protein can be produced as two separate proteins from an mRNA containing a single ORF, in which the translational regulator and fluorescent protein genes are paired via a self-cleaving peptide gene [2,23]. Since the translational level of these two proteins must be identical, the fluorescence signal can be used to monitor the expression level of the translational regulator in living cells. This method is particularly useful when the expression level of multiple proteins should be linked, but the function of each protein is affected if fused. However, it should be noted that after separation by this self-cleavage, both proteins are fused to fragments of the self-cleaving peptide, which may affect the properties of the proteins in some cases.

## 3. Optimization of RNA Motifs and Protein Modules

### 3.1. RNA Motif Optimization

**Figure 5:**
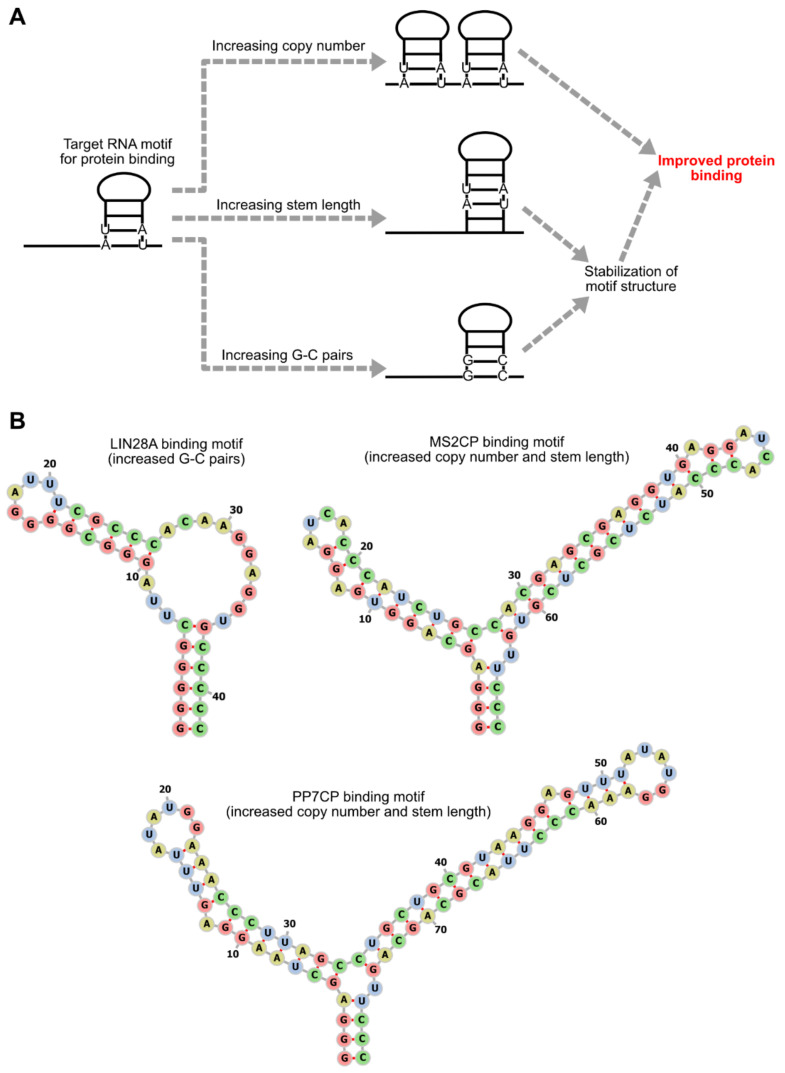
Enhancement of mRNA-protein interaction. (**A**) Approaches to enhance protein binding to mRNAs. Protein binding to mRNAs can be enhanced by increasing copy numbers or stabilizing secondary structures of target RNA motifs embedded in mRNAs. The secondary structure stabilization can be achieved by increasing stem length or G-C pairs. (**B**) Scheme 28. A [25], MS2CP[5], and PP7CP[19] are improved to enhance protein binding by approaches shown in (**A**). The sequences and the secondary structures of the original motifs are shown in Figure 3.

The copy numbers, sequences, and locations of RNA motifs that bind to translational regulator proteins have a large impact on the efficiency of translational regulation. One of the simplest ways to enhance the effects of regulator proteins is to increase the probability of regulator protein-binding to mRNAs by increasing the copy numbers of the target RNA motifs (Figure 5). Translational repression by binding of L7Ae or MS2CP to 5' UTRs was shown to become stronger as the copy numbers of the target motifs increase [11,21]. Similarly, the effects of the translational activator and the mRNA decay-promoting protein were also reported to increase as the copy numbers of target motifs in 3’ UTRs increase [19,33].

The efficiency of translational regulation can also be modulated by altering the sequence of RNA motifs. While L7Ae strongly represses the translation of mRNAs containing its target motif, kink-turn (K-turn), weaker translational repression can be obtained by using the K-loop motif, whose affinity to L7Ae is lower than K-turn [11,22]. For the MS2CP-target motif, the difference of the 3rd nucleoside in the loop region affects affinity. In the wild type MS2CP-target motif, the nucleoside at this position is uridine (U-variant), but the variant that has cytidine at this position (C-variant) has a higher affinity than the U-variant (Figure 3) [28], and this C-variant is now predominantly used in MS2CP-mediated translational repression. Stabilizing RNA secondary structures of target motifs through increases in stem region length or G-C pairs can also enhance protein binding and translational repression [5,21,27] (Figure 5). However, it should be noted that the presence of stable stem structures in 5' UTRs is itself disadvantageous for translation [34]. Thus, although the stabilization of target RNA motifs can improve the ON/OFF ratio, it may decrease the absolute amounts of protein production.

For mammalian applications of synthetic mRNAs, modified nucleosides (e.g., N1-methyl-pseudouridine) are often used to evade immune responses and increase translational efficiency [54,55,56]. These modified nucleosides also alter the affinity between translational regulator proteins and their target motifs, altering the efficiency of translational regulation. Parr et al. reported that MS2CP and U1A showed higher translational repression against mRNAs containing N1-methyl-pseudouridine than mRNAs containing uridine or pseudouridine [13]. Nevertheless, the use of modified nucleosides does not always improve protein binding. Wagner et al. reported that TetR has only a negligible doxycycline-responsive translational repression effect on N1-methyl-pseudouridine-containing mRNAs [23], although it can repress translation of mRNAs that contain no modified nucleosides [26].

In contrast to translational repression by RBPs, in the case of translational activation by CaVT, mRNAs containing a relatively weak affinity motif showed a higher response [5]. This could be explained by the balance between the effects of MS2CP and VPg, which are components of CaVT. If the affinity between MS2CP and the motif in the 5' UTR is too high, the translational repression effect of MS2CP may cancel out the translational activation effect of VPg. 

The locations of the target RNA motifs are also important in translational regulation. In the case of translational repression by protein binding to 5’ UTRs, it was reported that the closer the target motif is to the 5’ end of the mRNA, the higher the translational repression [11]. On the other hand, in the case of translational activation by CaVT, the highest translational activation was obtained when the target motif was located in the middle of the 5' UTR [5].

### 3.2. Protein Module Optimization

The affinity between RBPs and their target motifs can be modulated by introducing mutations in RBPs. For example, Stapleton et al. developed several L7Ae mutants with different affinities, although none of them showed higher translational repression than the wild type [22]. Recently, Fukunaga and Yokobayashi reported L7Ae mutants with different sequence selectivity, but their translational repression efficiency remains unknown [57]. 

In the case of MS2CP, mutants with higher translational repression efficiency (e.g., V29I mutant) have been reported. One such super-repressor mutant, termed dlFG, was produced by deleting the FG loop region of MS2CP. Since the FG loop is critical for MS2CP dimers to assemble into the capsid, deletion of the FG loop increases MS2CP dimer concentration by preventing capsid assembly and eventually enhances MS2CP dimer-mediated translational repression [58,59].

For U1A, a C-terminal deleted version is often used, as the N-terminal region of U1A is said to be an RNA binding domain. However, a recent study reported that the full-length U1A showed a higher translational repression efficiency than the C-terminal deleted version [21]. 

Goldfless developed a TetR mutant, named revTetR-S2, which has the opposite property of the wild type TetR [26]. While the wild-type TetR binds to its target RNA motif only in the absence of doxycycline, revTetR-S2 binds to the motif only in the presence of doxycycline.

## 4. Input Signals Utilized for Translational Regulation

### 4.1. Deliberate Regulation by External Cues

One approach to producing an optimal amount of protein is to deliberately control it with an external cue such as small molecule administration or light irradiation. In this case, one uses protein–protein interaction modules [4,5,6,14,20], degrons [4,6,20,23,25], or RBPs [23,26] that can be controlled by small molecules or light. These types of translational regulation systems allow for a gradual decrease or increase in translation by adjusting the dosage of a small molecule or the duration of light irradiation [20]. Thus, they enable not only simple on-off switching but also optimizing the level of translation. 

When the translation is planned to be regulated by an external cue, it is important to check the property of each cue and select the most appropriate one for application purposes. Light can reach the target cells immediately and enables not only temporal but also spatial regulation by irradiation to a specific site. On the other hand, light is not suitable to simultaneously regulate translation in the whole body. Particularly, irradiating cells in deep organs is a difficult task. Insertion of an optical fiber is one solution [60], despite being invasive. Red and near-infrared light have relatively higher tissue-penetrance [61], but there are relatively few proteins [62,63,64] that respond to such long-wavelength light. Most photo-controllable proteins respond to shorter wavelength light, such as blue light [51,65,66,67,68,69,70], although its tissue-penetrance is not high. Some nanoparticles may be useful to overcome the problem. For example, upconversion nanoparticles can convert near-infrared light into shorter wavelength light [71]. Mechanoluminescent nanoparticles are also promising, as they can emit light when they receive a focused ultrasound, which has higher tissue penetrance than light [72].

Small molecules such as doxycycline [23,26] or trimethoprim [4,6,20,23,25] are more suitable for whole body-translational regulation than light. Additionally, there are a number of small molecules that can be administered in a noninvasive manner like oral administration. On the other hand, spatially selective translational regulation by small molecules is difficult to achieve. Moreover, distinct from photo-regulation, in small molecule-mediated regulation, there is a time lag between the administration of the molecule and its arrival at the target tissues. There is also a time lag between the discontinuation of administration and the disappearance of the molecule from the body. These time lags make it difficult to rapidly change translation levels.

### 4.2. Endogenous or Pathogenic Protein-Responsive Autonomous Regulation

In cases where deliberate adjustment of the optimal translation level based on the physiological condition-monitoring is difficult, cell state-responsive autonomous translational regulation systems are well suited. Since the expression level of endogenous or pathogenic proteins depends on cell state, these proteins can be utilized as indicators of cell state to achieve cell state-responsive translational regulation. The simplest approach for detecting endogenous proteins with synthetic mRNAs is to add endogenous protein-targeting RNA aptamer sequences to the UTRs of synthetic mRNAs. This approach was previously used to distinguish pluripotent stem cells from differentiated cells by a LIN28A-binding aptamer-embedded synthetic mRNA, as LIN28A expression is high in pluripotent stem cells and low in differentiated cells [27]. Similarly, an alpha-fetoprotein-binding aptamer-embedded synthetic mRNA was utilized for the targeted killing of hepatocellular carcinoma cells [6].

Another approach is to detect the target protein not by synthetic mRNAs themselves, but by proteins expressed from them. Translational regulation systems that respond to Wnt, Myc, or viral NS3 protein are examples [4,12]. By combining the Wnt- and Myc-responsive systems, Yang and Ding succeeded in the selective killing of cells that were positive for both Wnt and Myc. The details of these systems are described in Section 2.4 and Section 2.6 (Figure 4).

### 4.3. microRNA-Responsive Autonomous Regulation

microRNAs (miRNAs) are also important indicators of cell state. miRNAs are small non-coding RNAs around 22 bases in length and function to repress the translation of mRNAs containing sequences that are complementary to miRNAs [73]. While there are more than 2600 miRNAs in human cells [74], their activities vary among cell types. Therefore, the activities of miRNAs to repress mRNA translation can be used as indicators of cell types [75]. 

To regulate the translation of synthetic mRNAs by miRNAs, complementary sequences of miRNAs that need to be detected are inserted into UTRs [2,3,5,13,75]. In cells lacking the corresponding miRNA activity, mRNAs containing miRNA complementary sequences are efficiently translated. In contrast, their translation is repressed in cells with high miRNA activity. The sensitivity of miRNA-responsive mRNAs depends on the copy numbers and the locations of the miRNA complementary sequences. In general, a higher copy number of the miRNA complementary sequence results in a more efficient translational repression [3,76], and the miRNA complementary sequence located in the 5' UTR is more efficient than that in the 3' UTR [3]. Moreover, translational repression was reported to be more efficient when the miRNA complementary sequence in the 5' UTR is located closer to the coding region [77].

**Figure 6:**
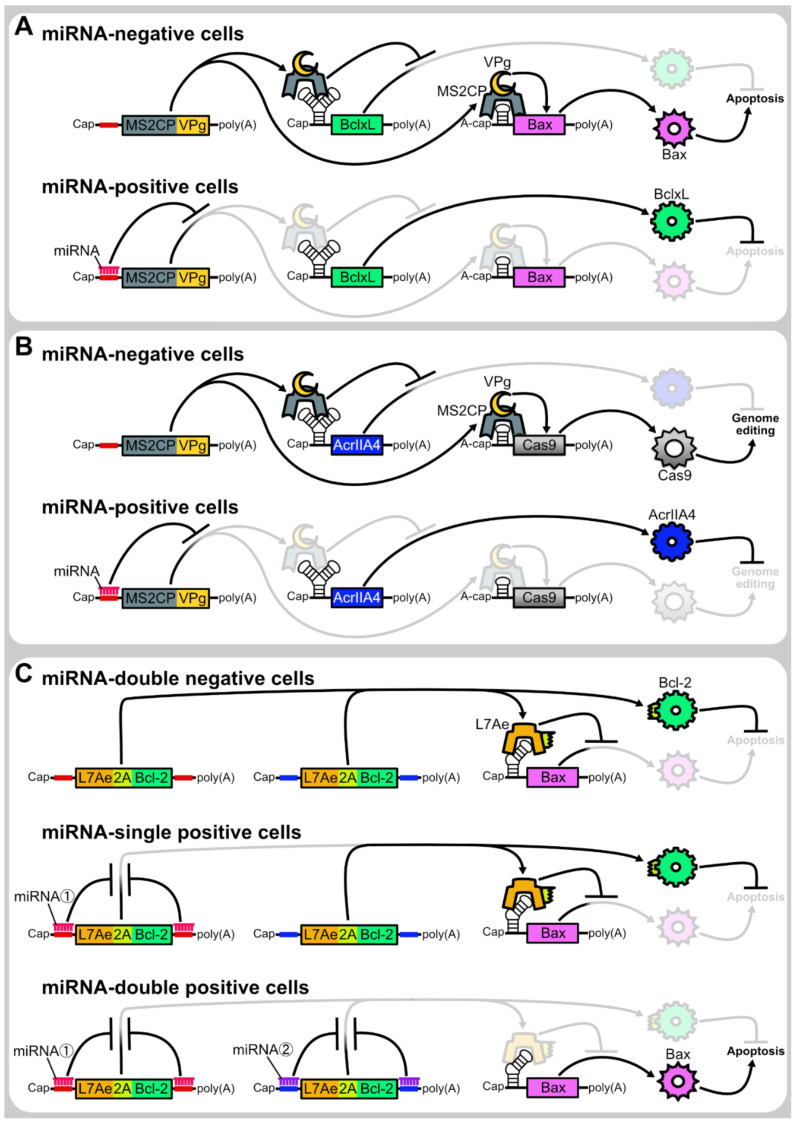
Representatives of microRNA-responsive translational regulation systems. (**A**) Apoptosis regulation by Caliciviral VPg-based Translational activator (CaVT). CaVT, which is composed of MS2CP and VPg, translationally represses the capped BclxL mRNA that contains a strong MS2CP binding motif. In contrast, the A-capped Bax mRNA containing a weak binding motif is translationally activated by CaVT. As BclxL is an anti-apoptotic protein and Bax is a pro-apoptotic protein, in miRNA-negative cells, apoptosis is induced by CaVT. In contrast, in miRNA-positive cells, CaVT is translationally repressed by the miRNA. Due to the repression of CaVT, the BclxL translation is not repressed and the Bax translation is not activated. BclxL inhibits the apoptosis which can be induced by the leaky expression of Bax. (**B**) The regulation of genome editing by CaVT. While CaVT translationally represses AcrIIA4 (an anti-CRISPR protein) mRNA, it translationally activates Cas9 mRNA. Thus, in miRNA-negative cells, genome editing is induced. In contrast, in miRNA-positive cells, CaVT is translationally repressed by the miRNA, which results in high expression of AcrIIA4 and low expression of Cas9. AcrIIA4 inhibits genome editing which can be induced by the leaky expression of Cas9. (**C**) Apoptosis regulatory AND circuit. In cells where either one of the two miRNAs is negative, L7Ae is translated and represses the Bax mRNA that contains a K-turn motif. Bcl-2 (an anti-apoptotic protein), which is simultaneously translated from the same mRNA as L7Ae, inhibits apoptosis which can be induced by the leaky expression of Bax. In contrast, in cells where both miRNAs are positive, L7Ae and Bcl-2 are translationally repressed. Due to the repression of L7Ae, Bax is efficiently translated and induces apoptosis.

Although miRNAs can be directly used to repress the translation of output proteins, the combination of translational regulator proteins and miRNA-responsive systems enables more complex and sophisticated regulation. For example, we developed a highly selective cell elimination system utilizing miRNA-responsive CaVT mRNAs. One distinct feature of CaVT is that it can be used for both translational activation and repression. While an mRNA containing a weak binding motif is translationally activated by CaVT, an mRNA containing a strong binding motif is repressed by it. Our miRNA-responsive cell elimination system consists of three mRNAs, a CaVT mRNA containing a miRNA complementary sequence, Bax (a pro-apoptotic gene) mRNA containing a weak binding motif, and BclxL (an anti-apoptotic gene) mRNA containing a strong binding motif. In miRNA-negative cells, CaVT translationally activates Bax and represses BclxL to induce apoptosis. In contrast, in miRNA-positive cells, both Bax activation and BclxL repression are abolished due to the miRNA-mediated repression of CaVT, which prevents apoptosis (Figure 6A). This system showed higher selectivity than the cell elimination by Bax-only regulation [5]. Similarly, we also achieved cell-selective genome editing by the simultaneous translational regulation of Cas9 and anti-CRISPR protein AcrIIA4 (Figure 6B). Another example is the apoptosis regulatory AND circuit developed by Matsuura et al. They designed two L7Ae mRNAs that have complementary sequences of two different miRNAs, miR-206 and miR-302a, respectively. They co-transfected these miRNA-responsive L7Ae mRNAs and a K-turn-embedded Bax mRNA, which is repressed by L7Ae. In cells containing both miR-206 and miR-302a, L7Ae-mediated repression of Bax is abolished, and eventually, apoptosis is induced. On the other hand, in cells lacking either miR-206 or miR-302a, L7Ae represses Bax and prevents apoptosis. Furthermore, to reduce the non-selective cell killing by the leaky expression of Bax, they linked the translation of L7Ae and Bcl-2 (an anti-apoptotic gene) (Figure 6C) [3].

## 5. Concluding Remarks and Future Perspectives

As described in this review, translational regulation systems based on RNA–protein interactions are well suited for the precise and complex regulation of synthetic mRNAs. In developing protein-based regulation systems, we can utilize and combine diverse proteins with different functions as modules. Furthermore, it is also possible to form multilayered gene circuits by utilizing output from one mRNA as input to regulate the other mRNA. Such multilayered gene circuits allow for even more complex regulation. Nevertheless, it is worth noting that synthetic mRNA-based gene circuits do not generally exhibit digital behavior like electronic circuits. In synthetic mRNA-based gene circuits, even in the translation-off state, there is often leaky expression, and the protein production is not completely zero. The effects of such leakage accumulate as gene circuit layers increase. Even such leaky expression alone can affect cells if the final output protein has a strong effect even in small amounts. On the contrary, if the final output protein needs very high amounts to affect the cells or organisms, it is possible that there will be no effect due to the shortage of absolute protein amount, even when there is a high fold-change from the off-state. Hence, high fold-changes observed in reporter gene assays do not always imply a high efficacy of the system in practical use. Thus, to put synthetic mRNA-based gene circuits for practical use, it is important to pay attention not only to fold-changes but also to absolute amounts of protein production. 

To develop synthetic mRNA-based gene circuits that achieve not only high fold-changes but also sufficient protein production, mRNAs should be carefully designed. For example, stabilization of target RNA motifs may improve fold-changes [21,27], but such stable RNA structures may also affect absolute amounts of protein production in a location-dependent manner. While stable RNA structures decrease protein production when they are placed in 5’ UTRs, they can increase protein production when they are placed in 3’ UTRs [34,78]. Similarly, although increasing the copy numbers or stem length of target RNA motifs in 5’ UTRs can improve fold-changes [11,21,27], it may decrease protein production, because too long 5’ UTRs are disadvantageous to translation [15]. Nucleoside modification is also an important factor that affects both fold-changes and absolute amounts of protein production. Several studies showed the superiority of N1-methyl-pseudouridine, a modified nucleoside used in COVID-19 vaccines [79], over other modified nucleosides such as pseudouridine and 5-methyl-cytidine [13,56,80]. However, the effects of nucleoside modifications on translation and immunogenicity depend on contexts such as mRNA sequences, cell types, and transfection reagents [78,81,82,83]. Therefore, to obtain the best performance in practical use, it is important to evaluate mRNAs in conditions as similar to practical conditions as possible.

While synthetic mRNAs enable the expression of any genes without the risk of insertional mutagenesis, the duration of gene expression from synthetic mRNAs tends to be shorter than that from DNAs, due to the short half-life of mRNAs [84]. To overcome the drawback, several methods have been developed, such as UTR optimization [85], mRNA circularization [86], and mRNA self-amplification [35,36]. Synthetic mRNAs that produce therapeutic proteins for long durations seem to be particularly useful in the treatment of genetic diseases such as cystic fibrosis and propionic acidemia. On the other hand, the long-term gene expression after a single administration also means that it is difficult to abolish protein production even when adverse effects are observed. The translational regulation systems for synthetic mRNAs are also useful to address such issues. 

There are many reports of mammalian gene circuits that are designed to treat diseases such as cancer, myocardial infarction, inflammatory disease, diabetes, and bacterial infection [4,6,7,87], although most of them were DNA-based. Currently, only limited types of proteins (e.g., fluorescent proteins and pro-apoptotic proteins) have been used as output of synthetic mRNA-based gene circuits. For further development of this field, applications of translational regulation systems to other therapeutic proteins, such as cytokines, antibodies, and reprogramming factors are also expected in the future.
